# Intraductal Papillary Mucinous Neoplasm and Pancreas Divisum: Two Cases

**DOI:** 10.1089/crpc.2016.0004

**Published:** 2016-04-01

**Authors:** Joseph A. Baiocco, Colin T. Ackerman, James L. Crawford, Charles J. Yeo

**Affiliations:** Department of Surgery, Jefferson Pancreas, Biliary, and Related Cancer Center, Thomas Jefferson University, Philadelphia, Pennsylvania.

**Keywords:** intraductal papillary mucinous neoplasms, pancreas divisum, uncinate process

## Abstract

**Background:** Pancreatic intraductal papillary mucinous neoplasms (IPMNs) are a subset of ductal cell tumors with potential for malignancy. Because it is difficult to predict whether and when they will become malignant, management and resection are widely debated.

**Case 1:** A 70-year-old male with a 1-year history of epigastric pain was found to have pancreas divisum with a dominant 2.4 cm multicystic uncinate process lesion communicating with the main pancreatic duct and associated uncinate duct dilation.

**Case 2:** An 83-year-old male with pancreas divisum had a 7.3 cm uncinate cystic lesion with mural nodularity that had increased in size from 2.1 cm in 2008.

**Conclusion:** Management of patients with IPMNs can be challenging and may require resection to prevent malignant transformation.

## Introduction and Background

Intraductal papillary mucinous neoplasms (IPMNs) of the pancreas are mucinous, cystic tumors originating from pancreatic ductal cells that have malignant potential. Precursor lesions can be categorized as low, moderate, or high-grade dysplasia (carcinoma *in situ*), or alternatively they may exhibit invasive carcinoma.^1,2^ Among patients with resected IPMNs, 40–60% have developed to invasive carcinoma, and these patients have 5-year postresection survival rates between 30% and 55%.^3,4^ Tumor markers typically used to diagnose pancreas cancer, such as cancer carbohydrate antigen (CA) 19–9 and carcinoembryonic antigen (CEA), are not uniformly elevated in IPMNs.

There are characteristic findings on radiological imaging that make high-risk IPMN more likely. These include main pancreatic duct dilation to >6 mm in diameter, cyst size >3 cm, and solid component (mural nodule) within a cyst, as per the Sendai consensus guidelines.^5^ A recent study has found uncinate duct dilation of at least 4 mm to be an additional risk factor for high-grade IPMN.^6^

Embryologically, the pancreas develops during the 6th or 7th week of gestation. The duodenal portion of the foregut gives rise to the dorsal and ventral pancreatic buds. The superior head, neck, body, tail, and accessory pancreatic duct are formed from the dorsal bud. The inferior pancreas head, uncinate process, and proximal main pancreatic duct arise from the ventral bud. During embryological week 8, the dorsal and ventral buds fuse to form the mature pancreas. When this occurs, the proximal dorsal accessory duct degenerates, and the remaining portion joins the ventral duct to form the main pancreatic duct. When this fusion does not take place, pancreatic divisum occurs such that the ventral duct becomes the main pancreatic duct (of Wirsung) and the dorsal duct becomes the accessory duct (of Santorini). In cases of pancreas divisum, the ventral duct drains to the major papilla, and the dorsal duct drains to the minor papilla.^7–9^

A small percentage of people with pancreas divisum develop symptoms due to relative stenosis of the orifice of the dorsal duct.^10^ As the scientific community's knowledge of IPMNs increases, there continues to be debate about when to manage with resection and when to manage with close observation. This question becomes more complicated in patients with anatomical abnormalities such as pancreas divisum.

This case report describes two patients with unusual IPMNs with uncinate duct dilation, which were originally managed with serial imaging. The patients elected to have resection of the neoplasm through pylorus-preserving pancreaticoduodenectomy (PPPD).

## Case Presentation 1

A 70-year-old man presented to the Jefferson Pancreas, Biliary, and Related Cancer Center for an opinion regarding surgical intervention for a pancreatic mass discovered 1 year earlier, during workup for epigastric pain, thought to be heartburn. He had no history of pancreatitis or obstructive jaundice. He also had no weight loss or steatorrhea. The patient did, however, develop new onset diabetes one year ago.

**FIG. 1.**
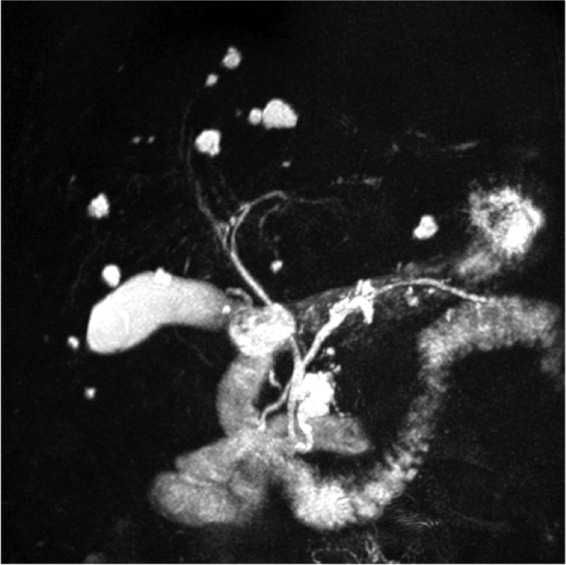
Coronal preoperative MRCP/MRI in patient 1 revealing pancreas divisum with a lobulated cyst lesion in the uncinate process as well as a notable dilation of the uncinate duct. Scattered benign hepatic cysts are noted, with a normal sized bile duct. MRCP, magnetic resonance cholangiopancreatoscopy.

Routine hematology and metabolic studies were normal. The tumor marker CA19-9 was slightly elevated at 41 U/mL (<35 U/mL) and the CEA was normal at 2.1 ng/mL (<4.7 ng/mL). An abdominal MRI with and without contrast revealed pancreas divisum with a dominant 2.4 cm multicystic uncinate process lesion communicating with the main pancreatic duct (duct of Wirsung) with an associated uncinate duct dilation to between 4 and 5 mm (Fig. 1). Imaging also showed simple hepatic cysts and minimal steatosis, but no evidence of metastasis. The lesion was believed to be an IPMN, based on this study and previous CT scans.

The patient underwent an open cholecystectomy and PPPD without complication. There was no evidence of malignant ascites, carcinomatosis, omental implants, or metastatic disease. Pathological study of the surgical specimen revealed the lesion to be an IPMN with intermediate-grade dysplasia without any invasive component. All surgical resection margins and 15 specimen lymph nodes were negative for malignancy.

The patient was placed on our institution's Whipple Accelerated Recovery Pathway (WARP) with the goal to discharge on postoperative day 5. However, he did develop a slight ileus, which prolonged his stay by 1 day. He was subsequently discharged on postoperative day 6. The patient and his family were appropriately educated on the postoperative recovery expectations, exercise recommendations, diet, and medications. He has fully recovered from his pancreatic resection and is scheduled to have annual surveillance of his pancreatic remnant through MRI/MRCP.

## Case Presentation 2

**FIG. 2.**
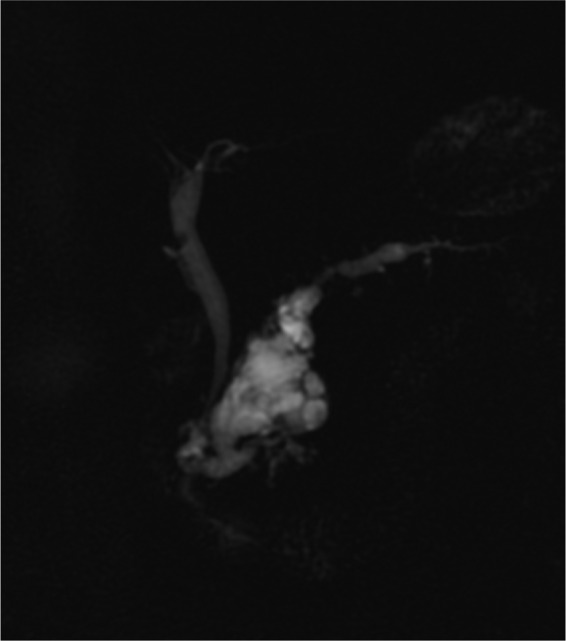
Coronal preoperative MRI in patient 2 revealing pancreas divisum with a cyst lesion measuring 7.3 cm involving the uncinate process and duct of Wirsung. Of note, mural nodularity is appreciated.

An 83-year-old male had an “incidentaloma” discovered in his pancreas on workup for autoimmune hepatitis. The patient was asymptomatic and denied ever having pancreatitis or obstructive jaundice. A cystic out pouching of the uncinate process duct measuring 2.1 by 1.8 cm was first discovered on MRI in 2008. Of note, pancreas divisum was also seen, with a dilated duct of Wirsung. No mural nodularity was appreciated. He was originally managed at an outside hospital where he had serial MRI studies, which showed only slight enlargement from 2008 to 2013. He had several samples of cyst fluid retrieved through endoscopic ultrasound, none of which generated a clear diagnosis of malignancy. He presented to the Thomas Jefferson Pancreas, Biliary, and Related Cancer Center in 2013 for a second opinion regarding the management of his indolent lesion. An MRI in April 2015 showed a mass consistent with mixed main duct and side branch IPMN measuring 3.2 cm in diameter. Subsequent MRI in October showed the cystic component of the mass had now increased to 7.3 cm with mural nodularity (Fig. 2).

The patient was taken to the operating room where he had a PPPD. The patient's postoperative course was uncomplicated, and he was discharged on postoperative day 8. The final pathology analysis of the specimen revealed IPMN w/high-grade dysplasia and pancreatic intraepithelial neoplasia, grade 3 (PanIN-3). There was extensive involvement of the secondary duct, but there was no evidence of invasion. Surgical resection margins and 18 specimen lymph nodes were negative for malignancy. He has fully recovered, returned to normal activities, and will be followed by annual MRI/MRCP to keep his remnant pancreas under surveillance.

## Discussion

IPMNs can be classified anatomically as main duct (MD-IPMN), branch duct (BD-IPMN), or mixed. The majority of MD-IPMNs arise in the pancreatic head, whereas most BD-IPMNs arise in the uncinate process.^11,12^

Although BD-IPMNs have a much lower rate of malignant transformation than MD-IPMNs (2–26% as compared with 70%), patient 1's previous good health made him an excellent surgical candidate.^6,13^ Although the lesion lacked the classical radiological findings that favor progression to malignancy, Ammori et al. recently reported that when uncinate duct dilation is greater than 4 mm, the chance of malignant transformation increases to 64%, regardless of anatomic classification.^6^

An additional factor that would help determine the likelihood of progression to malignancy in specific lesions is histological subtype. Gastric type IPMN, which expresses mucin-5AC and mucin-6, is the most common subtype overall and is associated with BD-IPMNs. Other subtypes—intestinal, pancreatobiliary, and oncocytic—are typically associated with MD-IPMNs.^14,15^ Gastric subtype has been found to have the lowest incidence of invasion at resection (31%).^15^ However, this is still a significant risk, especially considering that patient 1 had several factors shown to be associated with malignancy when the lesion is <3 cm: older age, male, and pancreatic duct dilation.^16^

Patient 2's case is notable for the rather rapid increase in size of the branch duct lesion, and for the pathological finding of high-grade dysplasia in the IPMN. The finding of high-grade dysplasia, also termed carcinoma *in situ*, causes one to exercise caution when recommending serial surveillance of the BD-IPMNs with uncinate duct dilation.

## Conclusion

We herein report two cases of BD-IPMNs located in the uncinate process, treated successfully by PPPD. On postoperative specimen pathology analysis, the lesions were found to contain intermediate and high-grade dysplasia. Due to the indolent course of BD-IPMNs, most lesions are followed by serial imaging. However, we suggest that lesions in the uncinate process that cause uncinate duct dilation should be considered a risk factor for high-risk/invasive IPMNs in addition to the previously described radiological findings. Further investigation is necessary to allow for better management of IPMNs.

## Abbreviations Used

BD-IPMN: branch duct-intraductal papillary mucinous neoplasm
CA: carbohydrate antigen
CEA: carcinoembryonic antigen
CT: computed tomography
IPMN: intraductal papillary mucinous neoplasm
MD-IPMN: main duct-intraductal papillary mucinous neoplasm
MRCP: magnetic resonance cholangiopancreatoscopy
PPPD: pylorus-preserving pancreaticoduodenectomy
